# The cancer-testis gene, *MEIOB,* sensitizes triple-negative breast cancer to PARP1 inhibitors by inducing homologous recombination deficiency

**DOI:** 10.20892/j.issn.2095-3941.2020.0071

**Published:** 2021-02-15

**Authors:** Yayun Gu, Cheng Wang, Rongxuan Zhu, Jianshui Yang, Wenwen Yuan, Yanhui Zhu, Yan Zhou, Na Qin, Hongbing Shen, Hongxia Ma, Hongxia Wang, Xiaoan Liu, Zhibin Hu

**Affiliations:** 1 State Key Laboratory of Reproductive Medicine, Center for Global Health, School of Public Health, Nanjing Medical University, Nanjing 211166, China; 2 Jiangsu Key Lab of Cancer Biomarkers, Prevention and Treatment, Jiangsu Collaborative Innovation Center for Cancer Personalized Medicine, Nanjing Medical University, Nanjing 211116, China; 3 Department of Bioinformatics, School of Biomedical Engineering and Informatics, Nanjing Medical University, Nanjing 211166, China; 4 Department of Oncology, Shanghai General Hospital, Shanghai Jiao Tong University School of Medicine, Shanghai 200080, China; 5 Department of Breast Surgery, The First Affiliated Hospital with Nanjing Medical University, Nanjing 210029, China

**Keywords:** Cancer-testis gene, *MEIOB*, triple-negative breast cancer, PARP1 inhibitor, cell proliferation

## Abstract

**Objective:**

The newly defined cancer-testis (CT) gene, *MEIOB,* was previously found to play key roles in DNA double-strand break (DSB) repair. In this study, we aimed to investigate the effects and mechanisms of MEIOB in the carcinogenesis of triple-negative breast cancers (TNBCs).

**Methods:**

The Cancer Genome Atlas database was used to quantify the expression of *MEIOB*. Cox regression analysis was used to evaluate the association between *MEIOB* expression and the prognosis of human TNBC. The effects of MEIOB on cell proliferation and migration in TNBCs were also assessed *in vitro*. Patient-derived xenograft (PDX) models were used to assess the sensitivity of breast cancers with active MEIOB to PARP1 inhibitors.

**Results:**

We confirmed *MEIOB* as a CT gene whose expression was restricted to the testes and breast tumors, especially TNBCs. Its activation was significantly associated with poor survival in breast cancer patients [overall, hazard ratio (HR) = 1.90 (1.16–2.06); TNBCs: HR = 7.05 (1.16–41.80)]. In addition, we found that *MEIOB* was oncogenic and significantly promoted the proliferation of TNBC cells. Further analysis showed that *MEIOB* participated in DSB repair in TNBCs. However, in contrast to its function in meiosis, it mediated homologous recombination deficiency (HRD) through the activation of polyADP-ribose polymerase (PARP)1 by interacting with YBX1. Furthermore, activated MEIOB was shown to confer sensitivity to PARP inhibitors, which was confirmed in PDX models.

**Conclusions:**

*MEIOB* played an oncogenic role in TNBC through its involvement in HRD. In addition, dysregulation of *MEIOB* sensitized TNBC cells to PARP inhibitors, so *MEIOB* may be a therapeutic target of PARP1 inhibitors in TNBC.

## Introduction

Triple-negative breast cancer (TNBC) accounts for approximately 10%–15% of all breast cancers and is defined as breast cancer that is negative for the estrogen receptor, progesterone receptor, and human epidermal growth factor receptor 2 (HER2)^1^. Due to the absence of these three receptors in TNBC, chemotherapy remains the standard-of-care for TNBC patients both in early and advanced stages^2,3^. Although chemotherapy can improve the conditions of patients with early TNBC, the recurrence and metastasis rate of TNBC patients is high for patients with advanced disease^4–6^. Thus, novel targeted approaches are urgently needed for the treatment of TNBC. Recently, olaparib, a polyADP-ribose polymerase (PARP) inhibitor, was approved by the US Food and Drug Administration for the treatment of breast cancers with *BRCA1/2* mutations^7^. *BRCA1/2* mutations in cancers are prototypic molecular alterations that confer homologous recombination deficiency (HRD) and sensitivity to DNA damaging therapy^8,9^. In addition, some studies show that cancers with genetic deficiencies involved in homologous recombination repair other than *BRCA* mutations, such as deficiencies in ATM, ATR, PALB2, and FANC, are also highly susceptible to PARP inhibitor treatment^10,11^. A group of cancer-testis (CT) genes is essential for homologous recombination^12,13^. These genes include the meiotic topoisomerase that catalyzes DNA double-strand breaks^14^, components of the synaptonemal complex (SYCP1)^15,16^, and multiple proteins that mediate homologue alignment or recombination (MEIOB)^17,18^. Luo et al.^17^ revealed that MEIOB is involved in highly ordered DNA doubled-strand break (DSB) repair during meiotic homologous recombination, by forming a complex with its cofactor, SPATA22. In our previous study, we identified *MEIOB* as a new CT gene involved in the carcinogenesis of lung cancer^19^. Nevertheless, its role and precise mechanism in TNBC remain unknown. We therefore characterized the involvement of MEIOB in the DNA repair process in TNBC patients, and further examined whether dysregulated MEIOB in TNBC conferred sensitivity to PARP inhibitors.

## Materials and methods

### Patient data

We determined the expression of *MEIOB* in breast cancer tissues by reanalyzing the raw RNA sequencing data of 1,058 patients from The Cancer Genome Atlas (TCGA) datasets. A standard STAR-HTSeq-DESeq2 pipeline was used to quantify gene expression^20^. We used normalized read counts > 5 as the cutoff to define the expression of *MEIOB*. The clinical information was obtained from Firehose Broad GDAC (http://gdac.broadinstitute.org/, version 2016_01_28). To define signature 3, we converted all mutation data from WGS datasets into a matrix (M) composed of 96 features comprising mutation counts for each mutation type (C>A, C>G, C>T, T>A, T>C, and T>G) using each possible 5′ and 3′ context for all samples, and applied the R package deconstructSigs12 to determine the proportion of each known mutational signature. The tissue array used in immunohistochemistry (IHC) analysis was obtained from Shanghai Outdo Biotech, Shanghai, China. This study was approved by the ethics committee of Nanjing Medical University (Approval No. 2015-SRFA-112), and all patients completed the Clinical Sample Informed Consent Form.

### Cell culture and cell line authentication

MDA-MB-231 and MDA-MB-468 cells were obtained from the American Type Culture Collection (Manassas, VA, USA), and the SUM1315MO2 cell line was provided by Stephen Ethier (University of Michigan), as previously described^21^. The cells were cultured in Dulbecco’s Minimal Eagle’s Medium (DMEM) (Gibco, Gaithersburg, MD, USA) containing 10% fetal bovine serum (FBS; Gibco) and incubated at 37 °C in 5% CO_2_ and saturated humidity. The transient overexpression or knockdown of *MEIOB* was performed using siRNA or *MEIOB* plasmids. The 3 cell lines were cultured in low glucose DMEM. All cell lines were cultured at 37 °C in a humidified chamber with 5% CO_2_, tested negative for mycoplasma (Lonza, Rockville, MD, USA), and were authenticated using short tandem repeat profiling within the last 3 years (FuHeng Biology, Xian, China).

### RNA isolation and qRT-PCR

Total RNA was extracted using TRIzol reagent (Thermo Fisher Scientific, Waltham, MA, USA) and reverse transcribed using the PrimeScript RT Reagent Kit (Takara, Mountain View, CA, USA). Expression of cDNA was quantified using the TaqMan Gene Expression Master Mix (Thermo Fisher Scientific) with an ABI 7900HT System (Applied Biosystems, Foster City, CA, USA). Primer sequences and their respective amplicon sizes for RT-PCR are summarized in **Supplementary Table S1**.

### Western blot analysis

The cells were washed 3 times with phosphate-buffered saline (PBS), and the total protein was isolated using protein lysis buffer. After centrifugation at 12,000 × *g* for 15 min at 4 °C, the cell debris was removed, and the supernatant (cell lysate) was used for Western blot. Protein concentrations were measured using a BCA assay (Beyotime, Beijing, China). Equal amounts of protein were separated by 10% SDS-PAGE and then transferred to polyvinylidene difluoride membranes (Millipore, Billerica, MA, USA). The membranes were blocked in blocking buffer (Tris-buffered saline, pH 7.6, 5% skim milk, and 0.05% Tween) at room temperature for 1.5 h. Then, the membranes were incubated at 4 °C overnight with primary antibody diluted in blocking buffer, followed by incubation with the corresponding secondary anti-IgG horseradish peroxidase conjugate (Santa Cruz Biotechnology, Santa Cruz, CA, USA) for 1.5 h. The antibody binding was visualized with ECL solution (Pierce Biotechnology, Rockford, IL, USA). The expression of proteins was assessed by immunoblotting and was normalized to that of glyceraldehyde 3-phosphate dehydrogenase (GAPDH). The antibodies were as follows: anti-GAPDH (KC-5G4; Kang Chen Tech, Shanghai, China), anti-MEIOB (ab178756; Abcam, Cambridge, MA, USA), anti- green fluorescent protein (GFP; 66002-I-Ig; Proteintech, Wuhan, China), anti-RAD51 (ab63801; Abcam), anti-YBX1 (ab76149; Abcam), anti-PARP1 (sc-8007; Santa Cruz Biotechnology), and anti-PAR (ALX-804-220-R100; Enzo Life Sciences, Farmingdale, NY, USA).

### Cell Counting Kit (CCK)-8 assay

CCK-8 was purchased from Dojindo Molecular Technologies (Kumamoto, Japan). Briefly, 2 h before each indicated time point, 10 μL of the CCK-8 solution was added to each well in a plate containing 100 μL of DMEM. Then, the absorbance at 450 nm was recorded using a microplate absorbance reader. Each count was determined as an average of 3 repeats, and each data point was the average of at least 3 experiments. All data were normalized to the control group.

### Colony formation assay

The colony formation assay of *MEIOB* knockdown or *MEIOB*-overexpressing MDA-MB-231 cells and *MEIOB*-overexpressing SUM1315MO2 cells was performed using 6-well dishes (500 cells/well), with 6 wells per condition. The number of colonies containing more than 50 cells was counted. Cells were fixed and then stained with Crystal Violet. Images were captured under a microscope, and 3 wells were counted by 2 independent investigators.

### Cell migration and invasion assays

Migration and invasion assays were performed using Transwell migration chambers (Corning, Corning, NY, USA). The cells were seeded at a density of 4 × 10^4^ cells/well. A volume of 100 μL of the cells was added to the upper chamber, while 600 μL of DMEM containing 10% fetal bovine serum was added to the lower chamber and incubated at 37 °C in 5% CO_2_. The cells attaching to the upper surface of the membrane were removed with a cotton swab, and the cells on the underside were fixed and stained with Giemsa (Dingguo Bio, Shanghai, China) for 3–5 min and counted (9 random fields) by 2 independent investigators. The results were normalized to those of the controls.

### Cell cycle analysis of tumor cells

The cell cycle was analyzed by flow cytometry. For cell cycle analysis, transfected lung cancer cells were suspended in 75% ethanol overnight and centrifuged at 1,000 rpm. The cell pellets were washed twice with PBS and re-suspended in PBS containing 50 mg/mL propidium iodide and 100 g/mL DNase-free RNase A. The cell suspension was incubated for 30 min at 37 °C and analyzed by flow cytometry. Cell cycle distribution was further analyzed with Cell Quest software (Becton Dickinson, San Jose, CA, USA) and Mod Fit LT (Verity Software House, Topsham, ME, USA).

### Immunohistochemical staining

Xenografts were formalin-fixed for at least 24 h and paraffin-embedded. Sections were evaluated by hematoxylin & eosin staining. IHC was performed on additional sections to detect MEIOB (ab178756; Abcam), PARP1 (sc-8007; Santa Cruz Biotechnology), and Ki67 (GB13030-2; Servicebio, Wuhan, China) following the manufacturers’ instructions.

### The DSB repair reporter assay for HRD

DSB repair efficiency was measured using a DSB reporter assay as previously described^22^. We are grateful to Prof. Mansour for providing vectors used in the DSB repair reporter assay. The GFP-based repair substrates were cloned using pEGFP-N1 (Invitrogen, Carlsbad, CA, USA) and pBluescriptII-KS (Stratagene, Amsterdam, The Netherlands) as backbones, with pGC for homology-directed GC. For the GC substrate, pGC, the 18-bp I-SceI recognition site was inserted into the unique BcgI site of pEGFP-N1, thereby inactivating the GFP-coding sequence. This intermediate, named pGC-intermedI, and linked to a modified fragment of pBluescriptII-KS, was generated as previously described^22^. To induce DSBs, SUM1315MO2 cells containing the stably integrated reporter construct for gene conversion of pGC were transfected with the I-SceI expression vector, pCMV3xnls-I-SceI (1 μg), using Fugene HD (Promega, Madison, WI, USA) as a transfection reagent. Forty-eight hours after transfection, the cells were assessed for green fluorescence using Western blot and flow cytometry (FACScan, BD Bioscience, San Jose, CA, USA).

### Immunofluorescence staining

For immunofluorescence staining, tissues were first washed in PBS 3 times to remove excess Optimal Cutting Temperature (OCT) buffer, and then they were blocked with goat serum for 30 min at room temperature. Then, they were incubated with primary antibodies at 4 °C overnight. Before incubation with secondary antibodies, the tissues were washed 3 times in PBS. The 4′,6-diamidino-2-phenylindole (DAPI) stain was simultaneously added with 2 antibodies (Alexa Fluor 488 and cyanine 3; Thermo Fisher Scientific) from different species for 2 h at room temperature. After washing the sections 3 times with PBS and mounting them with mounting medium, the images were acquired by confocal microscopy. The reagents were as follows: DAPI stain (G1012; Servicebio), anti-MEIOB (ab178756; Abcam), and anti-γ-H2AX (ab26350; Abcam).

### Patient-derived xenografts

Patient-derived xenografts were generated from primary or metastatic breast tumors using previously described procedures^23^. All animal procedures were reviewed and approved by the Institutional Animal Care and Use Committee and the Ethics Committee of Nanjing Medical University (Approval No. IACUC-1601117). We selected 6 of the established breast tumor samples, including 3 *MEIOB*-negative and 3 *MEIOB*-positive tumors, for further use in xenograft models.

### Statistical analysis

*MEIOB* gene expression data from TCGA data were analyzed for differential expression in tumor tissues *vs.* adjacent normal tissues. The association between *MEIOB* expression in all tumor tissue samples and survival was analyzed by Cox regression. Kaplan-Meier analysis was used to fit the survival curves, and significance was evaluated using the log-rank test. For experimental data, continuous values are described as the mean ± SE and tested by analysis of variance. A value of *P <* 0.05 was accepted as statistically significant. All statistical analyses of *in vitro* and *in vivo* assays were performed using Prism 6.01 (GraphPad, San Diego, CA, USA).

### Data availability

The data that support the findings of this study are available from the corresponding author upon reasonable request.

## Results

### MEIOB is expressed in TNBCs and is associated with poor survival in TNBC patients

**Figure 1:**
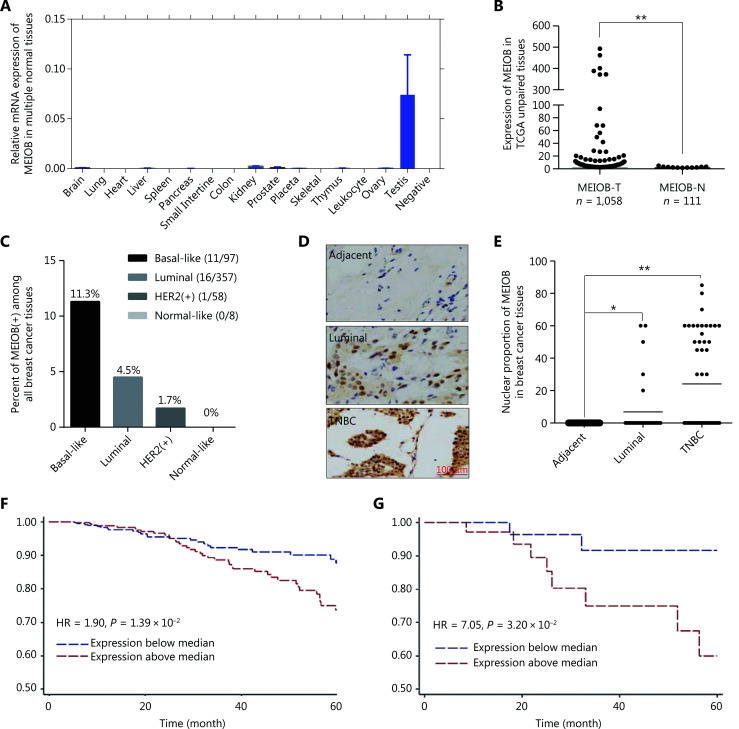
MEIOB is overexpressed in triple-negative breast cancers (TNBCs) and is associated with poor survival. (A) Expression of *MEIOB* in human adult normal tissues. RT-PCR was performed using the Human MTC™ Panel I kit. (B) *MEIOB* expression was higher in breast tumors than in adjacent tissues from The Cancer Genome Atlas (TCGA) database. **P < 0.01; *N*_tumor_ = 1,058, *N*_adjacent_ = 111. (C) The expression of *MEIOB* was significantly enriched in TNBC samples from TCGA. *N*_Basal-like_ = 97, *N*_Luminal_ = 357, *N*_Her2+_ = 58, and *N*_Normal-like_ = 8. (D and E) The results of immunohistochemical analysis in our breast cancer tissue array showed that the expression of MEIOB protein was significantly enriched in TNBCs. *P < 0.05; **P < 0.01; *N*_TNBC_ = 52, *N*_Luminal_ = 32, *N*_Adjacent_ = 32. (F) A Kaplan-Meier survival curve of breast cancer patients showed that higher expression of *MEIOB* in patients with breast cancer from TCGA was correlated with poorer overall survival; the Cox proportional hazards regression model of TCGA data was adjusted for tumor stage and menopausal status. (*n* = 1,050, *P* < 0.05). (G) Kaplan-Meier survival curve of TNBC patients showed that higher expression of *MEIOB* in patients with TNBC was correlated with poorer overall survival; the Cox proportional hazards regression model of TCGA data was adjusted for tumor stage and menopausal status (*n* = 98, *P* < 0.05).

To thoroughly evaluate the expression pattern of *MEIOB*, we conducted RT-PCR using cDNA of 16 normal human adult tissues, including the testis. The results showed that the expression of *MEIOB* in the testis was higher than that in any other normal tissue (**Figure 1A**), which was consistent with the results from the GTEx database. Furthermore, we found that *MEIOB* was aberrantly expressed in breast cancer tumor tissues based on TCGA data set analyses (**Figure 1B**), and the ratio of expression was the highest in TNBCs when compared with other subtypes of breast cancer (expression ratio: TNBC, 11.3%; luminal, 4.5%; HER2+, 1.7%; normal-like, 0%; **Figure 1C**). The results of IHC analysis of breast cancer tissues confirmed the upregulation of MEIOB protein levels in TNBCs when compared with both luminal cancers and adjacent tissues (*N*_TNBC_ = 52, *N*_Luminal_ = 32, *N*_Adjacent_ = 32; **Figure 1D and 1E**). In addition, Kaplan-Meier analysis showed that high *MEIOB* expression was positively correlated with poor overall survival in breast cancer patients, especially in TNBC patients from TCGA database [hazard ratio (HR) = 1.90 and 7.05, **Figure 1F and 1G**]. These findings suggested the tumor-promoting roles of* MEIOB* in the tumorigenesis and prognosis of breast cancer, especially TNBC.

### MEIOB alters cell viability in TNBCs

**Figure 2:**
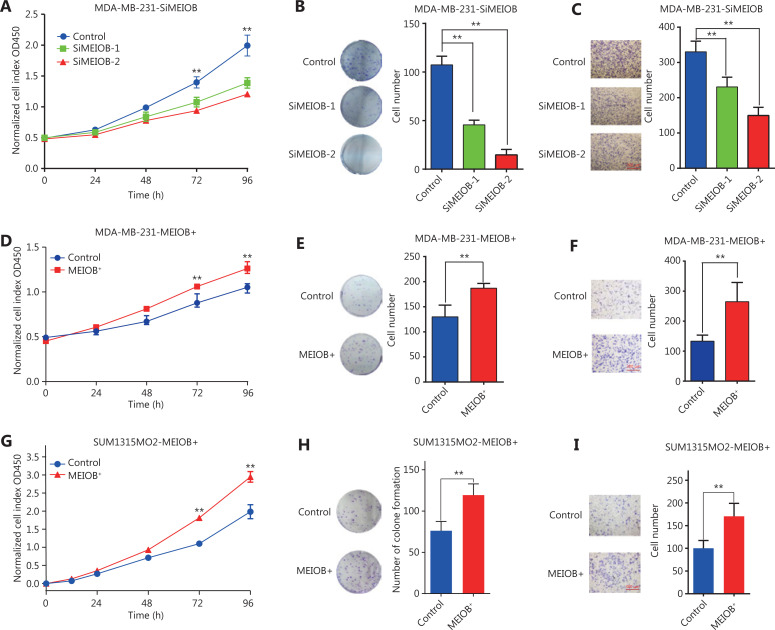
MEIOB is critical for triple-negative breast cancer (TNBC) cell proliferation and migration. (A) CCK-8 assays were conducted to determine the viability after knocking down MEIOB in MDA-MB-231 cells. Data are presented as the mean ± SEM, **P < 0.01. (B) Colony formation assays were conducted to determine proliferation after knocking down MEIOB in MDA-MB-231 cells. Data are presented as the mean ± SEM, **P < 0.01. (C) Transwell assays were conducted to determine metastasis after knocking down MEIOB in MDA-MB-231 cells. Data are presented as the mean ± SEM, **P < 0.01. (D) CCK-8 assays were conducted to determine the viability of MDA-MB-231 cells overexpressing MEIOB. Data are presented as the mean ± SEM, **P < 0.01. (E) Colony formation assays were performed to determine the proliferation of MEIOB-overexpressing MDA-MB-231 cells. Data are presented as the mean ± SEM, **P < 0.01. (F) Transwell assays were conducted to determine the metastasis of MEIOB-overexpressing MDA-MB-231 cells. Data are presented as the mean ± SEM, **P < 0.01. (G) CCK-8 assays were conducted to determine the viability of MEIOB-overexpressing SUM1315MO2 cells. Data are presented as the mean ± SEM, **P < 0.01. (H) Colony formation assays were performed to determine the proliferation of MEIOB-overexpressing SUM1315MO2 cells. Data are presented as the mean ± SEM, **P < 0.01. (I) Transwell assays were conducted to determine the metastasis of MEIOB-overexpressing SUM1315MO2 cells. Data are presented as the mean ± SEM, **P < 0.01.

To validate the hypothesis that MEIOB affected the viability of breast cancer cells, we first conducted RT-PCR and Western blot to examine the mRNA and protein expressions of MEIOB in various types of breast cancer cell lines. The results showed that both mRNA and protein levels of MEIOB were dramatically upregulated in two TNBC cell lines, MDA-MB-231 and MDA-MB-468, while they were significantly lower in either normal breast epithelium or luminal breast cancer cells (**Supplementary Figure S1A and S1B**), indicating that MEIOB might play an important role in a subset of TNBCs. Furthermore, immunofluorescence staining showed that MEIOB was localized in the nuclei of MDA-MB-231 cells (**Supplementary Figure S1C**). To better understand the role of MEIOB in breast cancer, we then knocked down MEIOB by using siRNA in MDA-MB-231 cells (**Supplementary Figure S2A and S2B**) and found that depletion of MEIOB in MDA-MB-231 cells led to a significant decrease in both cell proliferation and migration (**Figure 2A–2C**). We also overexpressed MEIOB in MDA-MB-231 cells (**Supplementary Figure S2C and S2D**), which significantly increased cell proliferation and migration (**Figure 2D–2F**). These results were further confirmed in SUM1315MO2 cells (**Figure 2G–2I**; **Supplementary Figure S2E and S2F**). Thus, we concluded that MEIOB may promote tumor proliferation in TNBCs.

### MEIOB induces homologous recombination deficiencies in TNBCs

**Figure 3:**
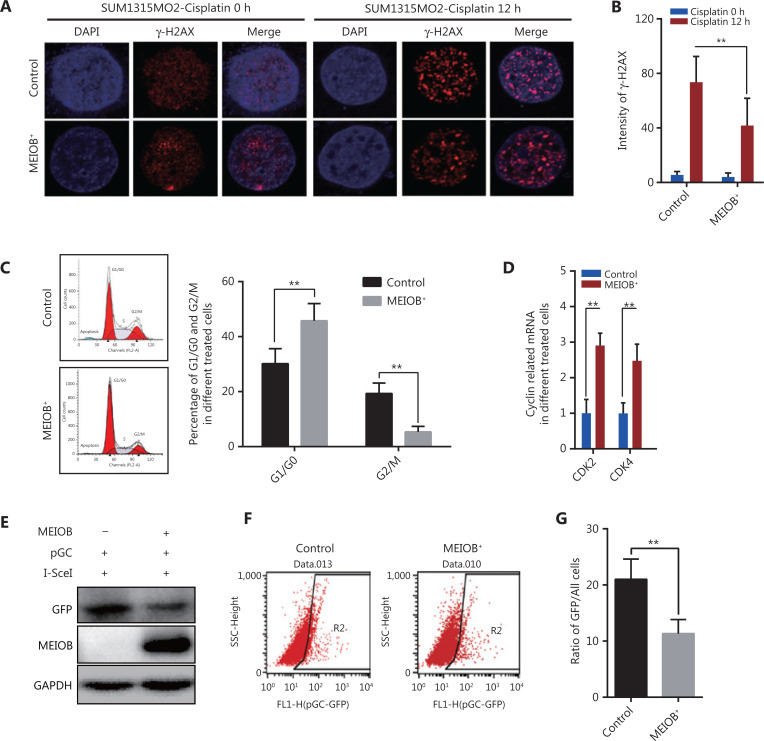
Overexpression of MEIOB induces homologous recombination defects. The formation of γ-H2AX foci decreases in MEIOB-overexpressing SUM1315MO2 cells. An immunofluorescence staining assay was conducted to examine the formation of γ-H2AX foci with 20 μM cisplatin treatment for 0 and 12 h. (A) Representative images of γ-H2AX foci. (B) Quantitative analysis of γ-H2AX foci per cell in each genotype from 3 independent experiments. *n* = 10 cells/groups. Data are presented as the mean ± SEM, **P < 0.01. (C) Cell cycle distribution in control and MEIOB-overexpressing SUM1315MO2 cells. Data are presented as the mean ± SEM, **P < 0.01. (D) The mRNA expression of key cell cycle regulators (CDK2 and CDK4) in control and MEIOB-overexpressing SUM1315MO2 cells. Data are presented as the mean ± SEM, **P < 0.01; *n* = 3/group. (E–G) Cells of different groups were transfected with pGC- and I-SceI-expressing vector, and the percentage of GFP^+^ cells (as an indication of homologous recombination efficiency) was assessed at the 72 h posttransfection time point by Western blot and flow cytometry. Data are presented as the mean ± SEM, **P < 0.01.

Cisplatin, a common chemotherapy agent that creates DNA interstrand and intrastrand crosslinks, can lead to DNA DSBs after its removal in both replicating yeast and mammalian somatic cells, and has been used to treat many breast cancer types, including TNBC^24–27^. We therefore treated SUM1315MO2 cells with cisplatin (20 μmol/L) to induce the DNA damage response and then measured the degree of DNA damage by monitoring anti-γ-H2AX nuclear staining signals^28^. The accumulation of γ-H2AX was significantly decreased in MEIOB-overexpressing SUM1315MO2 cells treated with cisplatin (**Figure 3A and 3B**), indicating that MEIOB promoted the DSB repair response in TNBC cells.

Homologous recombination is inhibited during the G1 phase of the cell cycle, but is active in both the S and G2 phases^29^. Our results further showed that the overexpression of MEIOB in SUM1315MO2 cells resulted in cell cycle arrest at the G1 phase, which indicated that MEIOB might be absent from homologous recombination while being involved in the non-homologous end joining (NHEJ) repair process (**Figure 3C**). As reported in past studies, CDK4 and CDK2 are necessary for cells to enter the G1 phase from the G0 phase^30^. In MEIOB-overexpressing SUM1315MO2 cells, we observed increased expressions of *CDK2* and *CDK4*, which further confirmed that MEIOB induced SUM1315MO2 cells to maintain G1/G0 phase arrest (**Figure 3D**). DSBs that occur during G1 phase are mainly repaired through NHEJ, whereas those formed during S and G2 phases are predominantly repaired by homologous recombination mechanisms^31^. Furthermore, the pHR-GFP reporter vector was used to assess the effect of MEIOB expression on DNA damage repair by homologous recombination in SUM1315MO2 breast cancer cells^32^. Homologous recombination activity was assessed by quantifying GFP protein expression and GFP-positive cells 72 h after co-transfection of pHR-GFP and pSce. As a result, we found that GFP intensity was significantly downregulated in MEIOB-overexpressing SUM1315MO2 cells when compared with control cells (**Figure 3E**). Similar results were observed in GFP-positive cells, as detected by flow cytometry (**Figure 3F and 3G**). Thus, we concluded that MEIOB overexpression in SUM1315MO2 cells resulted in HRD.

### MEIOB binds with YBX1 and activates PARP1-related repair

**Figure 4:**
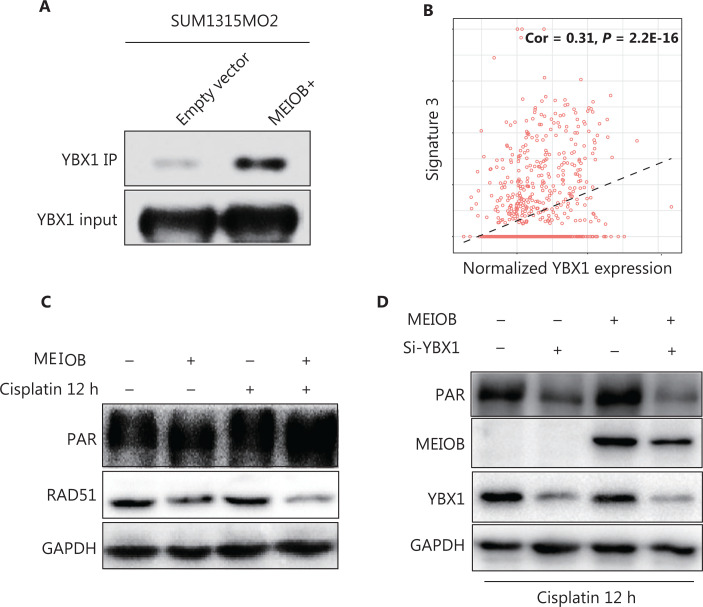
MEIOB binds with YBX1 and activates the PARP1 pathway. (A) FLAG-tagged MEIOB and vector controls were purified from MEIOB-overexpressing SUM1315MO2 cells and analyzed using mass spectrometry to identify MEIOB-binding proteins. YBX1 was found to bind with MEIOB in SUM1315MO2 cells. (B) The score of signature 3 was correlated with the expression of YBX1 in The Cancer Genome Atlas breast cancer datasets. Cor = 0.31, *P* < 0.001. (C) The expression of PAR was increased and the expression of RAD51 was decreased in SUM1315MO2-overexpressing cells after 20 μM cisplatin treatment for 12 h. (D) The increase in the expression of PAR in response to MEIOB overexpression was attenuated in SUM1315MO2 cells transfected with Si-YBX1 after 20 μM cisplatin treatment for 12 h.

To determine the mechanism by which MEIOB affected the DNA damage repair process, we performed liquid chromatography-tandem mass spectrometry to identify the proteins interacting with MEIOB. The results showed that the YBX1 protein, which can physically interact with PARP1 *in vitro* to inhibit PARG24 degradation of its polyADP-ribose, interacted with MEIOB (**Supplementary Figure S3A**, **Table S2**). This interaction was further verified by co-immunoprecipitation analysis of FLAG-tagged MEIOB (**Figure 4A**, **Supplementary Figure S3B**). We also found that the score of signature 3 in YBX1 high-expressing tumors was significantly higher than that in YBX1 low-expressing tumors in TCGA breast cancer datasets (**Figure 4B**). Next, we found that MEIOB caused an increase in PARP1 polyADP-ribose levels and a decrease in RAD51 expression, which is a key marker of the homologous recombination repair process (**Figure 4C**, **Supplementary Figure S3C**)^33^. Finally, the activated PAR-related DNA repair process in MEIOB-overexpressing cells was again silenced after YBX1 was knocked down (**Figure 4D**, **Supplementary Figure S3D**). Taken together, our results suggested that MEIOB was involved in PARP1-related HRD, probably by interacting with YBX1.

**Figure 5:**
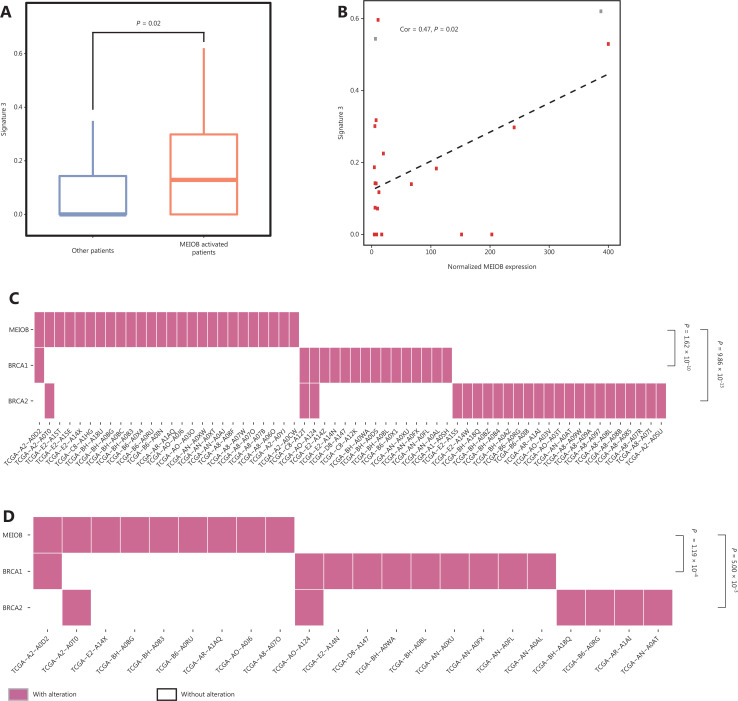
MEIOB expression is mutually exclusive with BRCA mutations. (A) The proportion of signature 3 was significantly higher in MEIOB-expressing patients than in non-expressing patients in The Cancer Genome Atlas (TCGA) breast cancer datasets. Student’s *t*-test was conducted, *P* = 0.02. (B) The score of signature 3 was correlated with the expression of MEIOB in TCGA breast cancer datasets. Cor = 0.47, *P* = 0.02. Red dots represent the BRCA-wildtype samples, and gray dots represent the BRCA-mutant samples. (C and D) Mutually exclusive pattern of MEIOB expression and BRCA1/2 mutation in breast cancer. *N*_overall_ = 459, *P* < 0.001 (C); *N*_TNBC_ = 75, *P* < 0.001 (D).

Biallelic inactivation of BRCA1/2 is associated with a pattern of HRD known as signature 3^34^. According to the results described above, we hypothesized that MEIOB might result in genomic alterations similar to the loss of BRCA1/2. Notably, we found that the score of signature 3 was correlated with MEIOB and was significantly higher in patients with high MEIOB expression in TCGA breast cancer datasets (**Figure 5A and 5B**). To exclude the influence of BRCA1/2, we found that MEIOB expression was mutually exclusive with mutations in BRCA1/2 in both breast cancers and TNBCs (*P* < 0.01, **Figure 5C and 5D**). These results suggested that MEIOB might be a new biomarker of HRD in TNBC patients, except in patients with BRCA1/2 mutations.

### MEIOB increases the sensitivity of TNBCs to the clinical PARP1 inhibitor, olaparib

**Figure 6:**
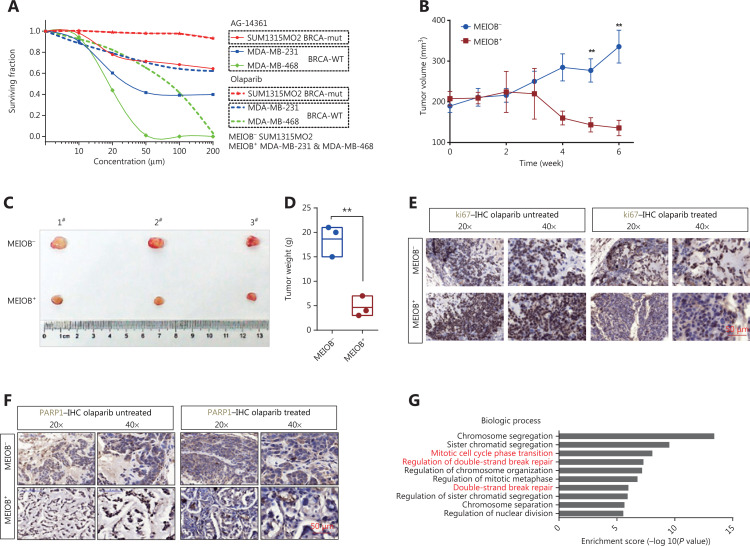
MEIOB increases the sensitivity of cancers to PARP1 inhibitors. (A) Dose-response PARP1 inhibitor survival curves for 3 breast tumor cell lines, SUM1315MO2, MDA-MB-231, and MDA-MB-468. The cells were plated in 96-well plates and constantly exposed to inhibitors for 3 days, at which point survival was estimated. (B) Curves showing the development of breast cancer cells from patient-derived xenografts treated with olaparib. Data are presented as the mean ± SEM, **P < 0.01. (C and D) Representative images and weight analyses of tumors harvested at the end point. Data are presented as the mean ± SEM, **P < 0.01. (E and F) Ki67 and PARP1 levels of tumors treated with olaparib were evaluated by immunohistochemical analysis. (G) GO analysis of significantly up/downregulated genes in olaparib-treated tumors.

We hypothesized that TNBC cells overexpressing MEIOB would be more sensitive to PARP inhibitors than MEIOB-null cells. We evaluated the sensitivity of TNBC cells with different MEIOB expression patterns to PARP1 inhibitors, by treating cells with multiple concentrations of the PARP1 inhibitors, AG-14361 and olaparib. **Figure 6A** shows that MEIOB-expressing MDA-MB-231 and MDA-MB-468 cells were more sensitive to AG-14361 than MEIOB-null SUM1315MO2 cells. Similar results were observed in cells treated with olaparib.

We then established a patient-derived xenograft (PDX) mouse model of TNBC that had characteristics similar to those of human primary tumors. A schematic of the PDX model is shown in** Supplementary Figure S4A**. Fresh TNBC tissues were obtained from patients and divided into two groups according to MEIOB protein expression levels (MEIOB-null and MEIOB-expressing) (**Supplementary Figure S4B**). All tumors were implanted subcutaneously in NOD/SCID mice. Both groups were treated with olaparib when the patient-derived tumor volume had reached 200 mm^3^. Tumor volumes were measured once a week after treatment with olaparib (**Figure 6B**). Six weeks after administration, the tumors were harvested and weighed (**Figure 6C**). The results showed that the volumes of tumors expressing MEIOB significantly decreased to less than 30% of MEIOB-null tumors after treatment with olaparib (**Figure 6D**), suggesting that MEIOB-expressing tumors, *in vivo*, were more sensitive to olaparib than MEIOB-null tumors.

In olaparib-treated PDX tumors, IHC labeling was conducted using antibodies to cell proliferation markers, Ki67 and PARP1. After treatment with olaparib, the number of Ki67-positive proliferating cells was dramatically decreased in the MEIOB-expressing tumors when compared with the MEIOB-null tumors (**Figure 6E**). In addition, PARP1 was strongly inhibited by olaparib in MEIOB-expressing tumors (**Figure 6F**). These results indicated that tumors that expressed elevated levels of MEIOB were more sensitive to olaparib.

To validate the downstream mechanisms of olaparib treatment, RNA-seq was performed with the olaparib-treated and untreated MEIOB-expressing tumors. We then selected differentially expressed genes for further Gene Ontology (GO) analysis. The results confirmed that olaparib therapy led to an accumulation of DNA DSBs and cell cycle arrest (**Figure 6G**)^35,36^. Representative gene expression levels involved in the DNA repair process were then verified by RT-PCR. **Supplementary Figure S4C** shows that expressions of the *PARPBP*^37^, *POLQ*^38^, and *RTEL1*^39^ genes, which participate in NHEJ, were inhibited by olaparib. A previous study showed that olaparib monotherapy provided a significant benefit over standard therapy in breast cancers with BRCA mutations^7^. Taken together, these results suggested that PARP inhibitors may have additional applications in the treatment of TNBC.

## Discussion

It has long been acknowledged that the processes of germ cell development and tumorigenesis share important similarities, including meiosis during gametogenesis and aneuploidy during tumorigenesis^12^. *MEIOB* is a newly identified CT gene that is expressed both in the testes and in tumor tissues from TNBC patients^19^.

TNBC is the most aggressive subtype of breast cancer^40^ and has no specific treatment guidelines^41^. It is generally thought that some TNBCs are susceptible to DNA damaging agents due to a dysfunctional DNA repair system. Several studies have shown that PARP inhibitors might be a promising therapeutic strategy for TNBC patients with BRCA1/2 mutations^42,43^, which can lead to the failure of double strand break repair, accumulation of genomic instability, and eventual tumor cell death^44^. However, TNBCs involve a heterogeneous group of breast cancers, and many TNBC patients do not have BRCA1/2 mutations^45^. A growing number of studies have suggested that sporadic TNBCs with genetic deficiencies other than BRCA1/2 generally bear signature 3, the marker of HRD, and are highly susceptible to PARP inhibitors^34^. We found that sporadic *MEIOB*-expressing TNBC patients bear substantial mutation signature 3 expressions, using multi-omics analyses of TCGA database. In addition, its expression was mutually exclusive with BRCA1/2 mutations. Furthermore, our results both *in vitro* (cells) and *in vivo* (PDX models) showed that the overexpression of MEIOB increased the sensitivity of TNBC to PARP1 inhibitors in patients without BRCA1/2 mutations. Thus, it is possible that MEIOB dysregulation might be considered a biomarker for the selection of TNBC patients that could be responsive to PARP inhibition.

In this study, MEIOB was involved in the error-prone NHEJ repair process in TNBCs, which was characterized by an increase in PAR and a reduction in RAD51^46^. In contrast, it was reported that MEIOB binds with SPATA22^17^ and plays key roles in the early repair of the meiotic homologous recombination repair process^18^. This suggested that MEIOB plays different roles during two biological processes; MEIOB strongly activates the homologous recombination repair pathway in meiosis^18^; and in a different manner, it induces another major DSB repair pathway, error-prone NHEJ, and results in HRD in TNBC. In TNBCs, MEIOB binds to YBX1, a protein that physically interacts with PARP1 to inhibit its polyADP-ribose degradation^47^. YBX1 was previously found to interfere with PARP1 activation on the damaged DNA by multimerization or interaction with its DNA-binding domain^48^. Furthermore, we observed an increase in PAR caused by MEIOB, which was attenuated after YBX1 was knocked down. These results led us to hypothesize that YBX1 may function as a cofactor of MEIOB in the synthesis of PAR.

## Conclusions

Taken together, our study showed that the CT gene, MEIOB, promoted breast cancer cell viability and mediated HRD by activating the PARP1-dependent DNA damage response. Additionally, MEIOB sensitized TNBC to PARP1 inhibitors, which might expand the use of PARP1 inhibitors for the treatment of MEIOB-positive TNBCs (**Supplementary Figure S4D**).
