# Supplementary material for: The cancer-testis gene, MEIOB, sensitizes triple-negative breast cancer to PARP1 inhibitors by inducing homologous recombination deficiency
Source: Cancer Biol Med. 2021 Feb 15;18(1):74–87. doi: 10.20892/j.issn.2095-3941.2020.0071 (PMC7877187; doi:10.20892/j.issn.2095-3941.2020.0071)
Supplement: Supplementary file 1 [file cbm-18-074-s001.pdf]

Supplementary materials

Table S1 Primers involved in TaqMan and PCR reactions using PCR

Gene name	Primer sequence
MEIOB-Forward	5′GACTGATCACACAGGCACCC
MEOB-Reverse	3′-ATTGCAAGAAACTCATGTACCGTG
CDK2-Forward	5′-TGGCGCTTAAGAAAATCCGC
CDK2-Reverse	3′-GCGAGTCACCATCTCAGCAA
CDK4-Forward	5′-AATGTTGTCCGGCTGATGGA
CDK4-Reverse	3′-GTGCCATCTGGTAGCTGTAGA
PARPBP-Forward	5′-AAGGCCAAAACAGCAGGGAT
PARPBP-Reverse	3′-GCTGCTTCTTCGTCCAAAAGA
POLQ-Forward	5′-CCCTGTACCGCTTTTGGAGT
POLQ-Reverse	3′-TGATATCTGCCAGCTTCTCACA
RTEL1-Forward	5′-TCCTTTGACCTGACTCCCCA
RTEL1-Reverse	3′-GCCCTTGGTCTGAAACGTGA
GAPDH-Forward	5′-TGCACCACCAACTGCTTAGC
GAPDH-Reverse	3′-GTCTTCTGGGTGGCAGTGATG

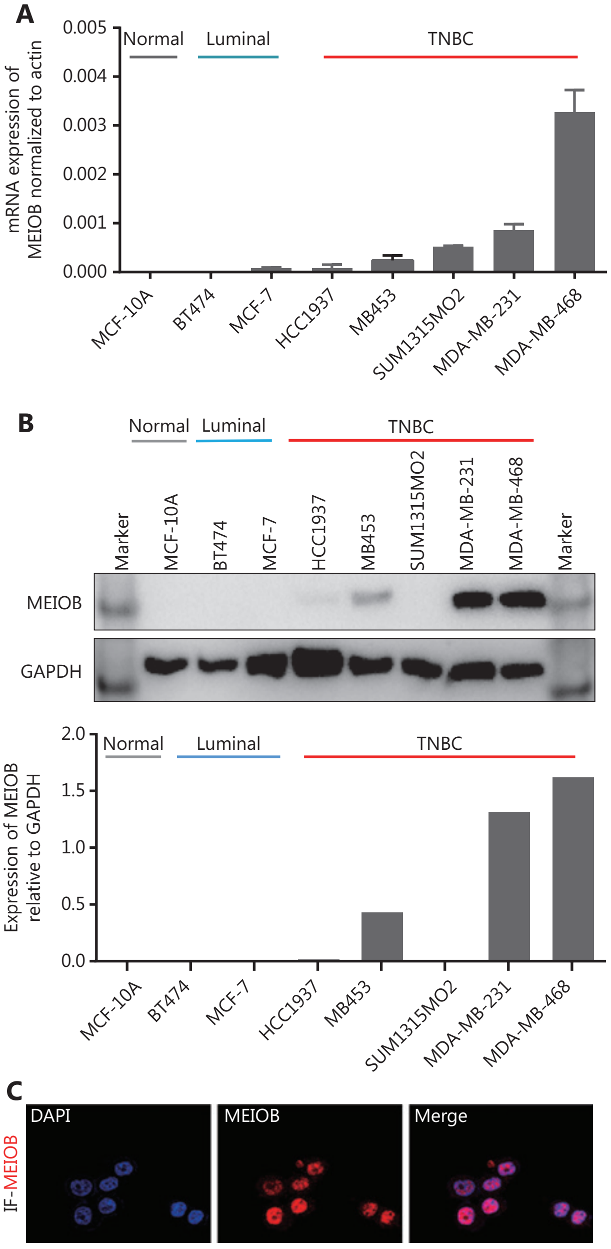

Figure S1 Expression of MEIOB in multiple breast cancer cell lines. (A and B) The mRNA (A) and protein (B) expressions of MEIOB were significantly higher in MDA-MB-231 and MDA-MB-468 cells than other cell lines. The protein expression of MEIOB relative to glyceraldehye 3-phosphate dehydrogenase was quantified by ImageJ software. (C) Immunofluorescence analysis of MEIOB localization in MDA-MB-231 cells (red, MEIOB; blue, nuclei), showed that the MEIOB protein was located in the nucleus.

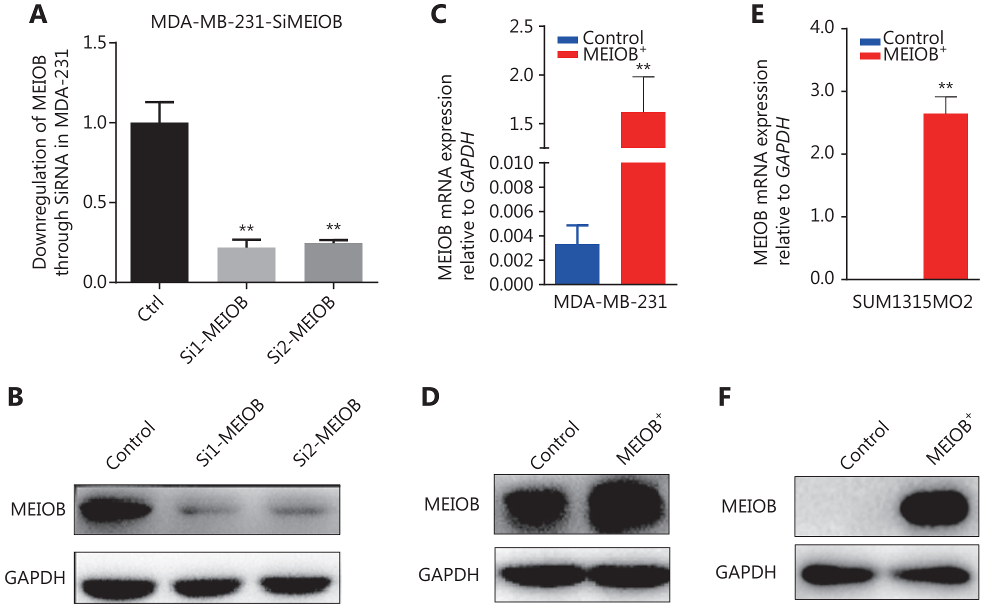

Figure S2 Validation of MEIOB expression in knockdown and overexpressed cells. (A and B) MEIOB was knocked down in MDA-MB-231 cells by siRNAs and was verified by RT-PCR (A) and Western blot (B). (C–F) MEIOB was overexpressed in MDA-MB-231 cells. The mRNA expression is shown in (C) and protein expression is shown in (D) and SUM1315MO2 cells. The mRNA expression is shown in E and protein expression is shown for all data and are expressed as the mean ± SEM, $^{**}P < 0.01$.

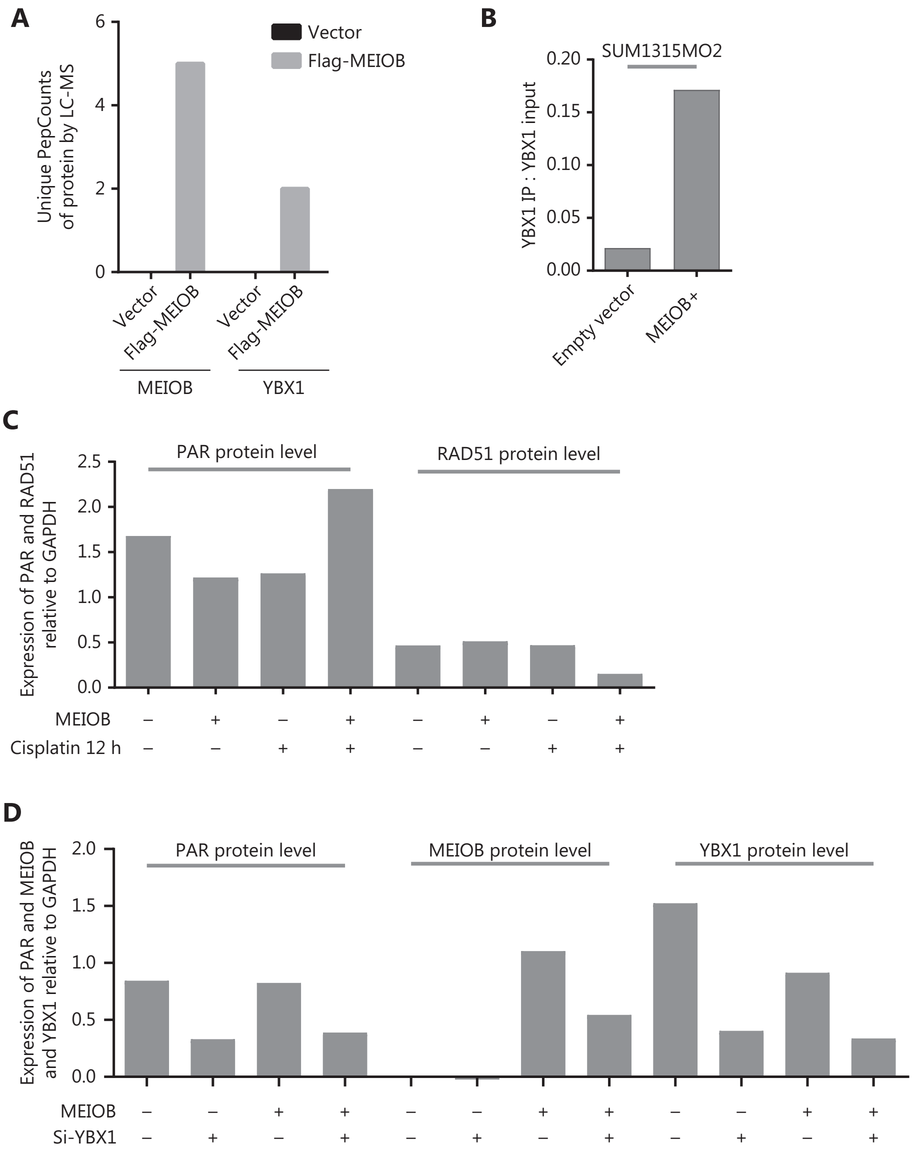

Figure S3 MEIOB binds with YBX1 and activates PARP1-related repair. (A) Liquid chromatography-tandem mass spectrometry for MEIOB and YBX1. Unique peptide counts of MEIOB and YBX1 in SUM1315MO2 cells based on co-immunoprecipitation assays and mass spectrometry analyses. (B) The protein expression of YBX1 binding to MEIOB relative to glyceraldehyde 3-phosphate dehydrogenase was quantified by ImageJ software. (C) The quantified protein expression of PAR and RAD51 in overexpressed SUM1315MO2 cells after 20 μM cisplatin treatment for 12 h. (D) The quantified protein expression of PAR, MEIOB, and YBX1 in overexpressed SUM1315MO2 cells transfected with Si-YBX1 after 20 μM cisplatin treatment for 12 h.

Table S2 A list of differential MEIOB-interacting protein candidates involved in DNA repair processes based on co-immunoprecipitation assays and mass spectrometry analyses

UniProt ID	Gene name	UniquePep Counts		Cover percent (%)	Length of AA	Reference for DNA repair
		MEIOB-tag	Control			
Q8N635	MEIOB	5	0	11.76%	442	24240703,26520106
B2RDN9	G22P1	2	0	3.94%	609	29247009,28959970
E9PHA6	MSH2	2	0	2.17%	934	27590317,29892060
Q6PKI6	YBX1	2	0	13.53%	324	27667193,14718551

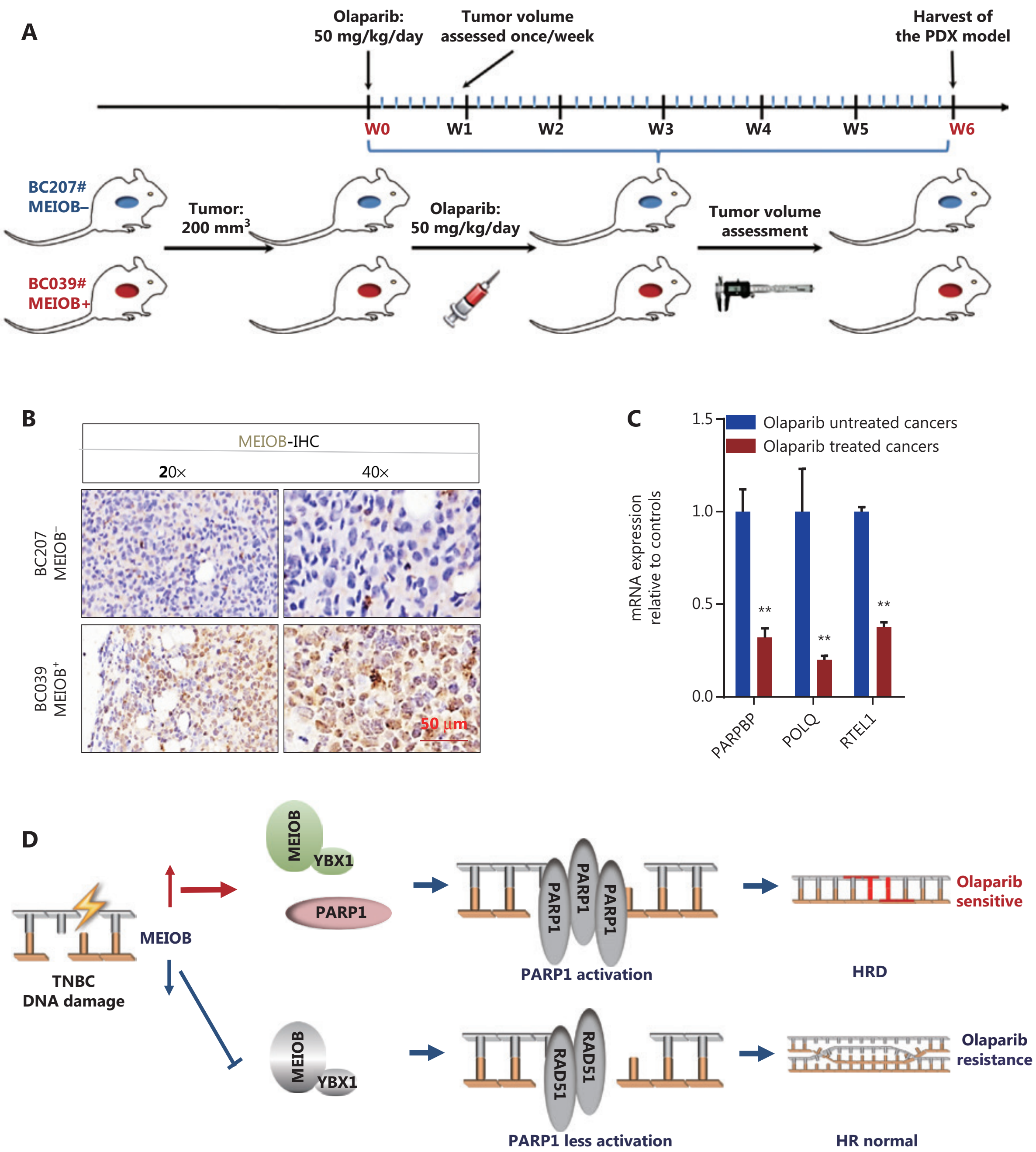

Figure S4 MEIOB sensitizes TNBC to PARP1 inhibitors in patient-derived xenograft (PDX) models. (A) Schematic of the PDX model. We divided all the tumor tissues into 2 groups according to MEIOB expression levels ($MEIOB^+$ and $MEIOB^-$). Both groups were treated with olaparib when the tumor volume had reached 200 mm^3. Tumor volumes were measured once a week. Six weeks later, the tumors were harvested and weighed. (B) MEIOB protein levels of patient-derived xenografts were evaluated by immunohistochemical analyses. (C) RT-PCR analysis was performed to verify the signaling identified by Gene Ontology analysis. Data are expressed as the mean ± SEM, n = 3/groups, $^{**}P < 0.01$. (D) Graphic abstract shows the tumor-promoting role of MEIOB binding with YBX1 and sensitizing the triple-negative breast cancers to the PARP1 inhibitor.
